# Zhuanggu Shubi ointment mediated the characteristic bacteria-intestinal mucosal barrier-bone metabolism axis to intervene in postmenopausal osteoporosis

**DOI:** 10.3389/fcimb.2024.1500111

**Published:** 2024-12-04

**Authors:** Xiaoya Li, Ning Li, Huan Pei, Yu Ren, Lei Li, Lan Sun, Yueying Wu, Jiali Yuan, Yuan Ma

**Affiliations:** ^1^ School of Basic Medical Sciences, Yunnan University of Chinese Medicine, Kunming, Yunnan, China; ^2^ Yunnan Provincial Key Laboratory of Integrated Traditional Chinese and Western Medicine for Chronic Disease in Prevention and Treatment, Yunnan University of Chinese Medicine, Kunming, Yunnan, China; ^3^ Department of Orthopedicis, The Third People’s Hospital of Yunnan Province, Kunming, Yunnan, China

**Keywords:** postmenopausal osteoporosis, Zhuanggu Shubi ointment, characteristic bacteria, bone formation, bone resorption, intestinal mucosal barrier, intestinal inflammatory

## Abstract

**Background:**

Zhuanggu Shubi ointment (ZGSBG) has good efficacy in postmenopausal osteoporosis (PMO), but the mechanism of efficacy involving gut microecology has not been elucidated.

**Objective:**

This study investigated the mechanism of ZGSBG in regulating gut microecology in PMO.

**Methods:**

The bilateral ovarian denervation method was used to construct a rat model of PMO and was administered ZGSBG. Behavior, bone transformation, gut microbiota, intestinal mucosal barrier, and intestinal inflammatory-related indexes were detected.

**Results:**

After ZGSBG intervention, bone R-hydroxy glutamic acid protein and procollagen type I N-terminal propeptides were significantly upregulated, while C-terminal telopeptide of type-I collagen and tartrate-resistant acid phosphatase-5b were significantly downregulated. Pathological analysis demonstrated an improvement in femoral and colonic structures. The expressions of zonula occludens-1, occludin, claudin-1, and secretory immunoglobulin A in the colonic tissues were significantly elevated, while the levels of tumor necrosis factor-α, interleukin-1β, interleukin-6, and lipopolysaccharides were reduced. Moreover, characteristic bacteria *Muribaculaceae* and *Prevotella* were significantly enriched. Furthermore, *Muribaculaceae* and *Prevotella* have a positive correlation with intestinal mucosal barrier function and a negative correlation with intestinal inflammatory responses.

**Conclusion:**

ZGSBG promoted bone formation, inhibited bone resorption, regulated gut microbiota, repaired intestinal mucosal barrier damage, and inhibited intestinal inflammatory responses in PMO rats. *Muribaculaceae* and *Prevotella* might play positive roles in ZGSBG treatment of intestinal mucosal barrier injury and inflammatory reactions in PMO.

## 1 Introduction

Postmenopausal osteoporosis (PMO) is a systemic metabolic bone disease characterized by a decrease in bone mass, degradation of the bone microstructure, and an increased risk of fracture, which is most often caused by estrogen deficiency in women after menopause (Chinese Society of Osteoporosis and Bone Mineral Research, Guidelines for the Diagnosis and Treatment of Primary Osteoporosis., 2023; Hu et al., 2022). It has been reported that the elderly population in China is gradually increasing and the degree of aging is deepening (Lu and Liu, 2021). The prevalence of PMO over 50 years of age is 32.1%, far exceeding that of European countries and the United States of America, and the prevalence gradually increases with age (Chinese Society of Osteoporosis and Bone Mineral Research, Epidemiological Survey on Osteoporosis in China and Results of the “Healthy Bones” Special Campaign Released., 2019). The high disability and mortality rates caused by PMO affect the physical and mental health of patients and also place a burden on society (Jayusman et al., 2023). Currently, estrogen, selective estrogen receptor modulators, and bisphosphonates are used in the clinical treatment of PMO, but long-term use can cause gastrointestinal discomfort, acid reflux, and other adverse symptoms. Furthermore, the drugs are expensive and need to be taken for a long time (Reid, 2015; Sambrook et al., 2010). Hence, it is particularly important to seek safe and effective treatments to improve PMO symptoms.

With the continuous deepening of research, scholars have gradually realized that the change in estrogen level is not the only factor in the occurrence and development of PMO, and various factors such as gut micro-environmental disorders, intestinal mucosal barrier damage, and inflammatory responses are also involved (Zhang., 2019; Lei et al., 2021). Under normal circumstances, the homeostasis of human bone mass can be achieved by regulating osteogenesis and the function of osteoclasts. Due to the decrease of estrogen levels in postmenopausal women, a structural disorder of gut microbiota is induced, the permeability of the intestinal mucosal barrier is increased, and the toxins released by pathogenic bacteria enter the bloodstream through the damaged barrier, inducing inflammation and causing the occurrence of PMO (Yang et al., 2022; Xu et al., 2022; Rettedal et al., 2021; Jia et al., 2017). Thus, PMO is closely related to gut microbiota, intestinal mucosal barrier function, and inflammatory responses.

Zhuanggu Shubi ointment (ZGSBG) was formulated by Prof. Li Qingsheng, a famous Chinese medicine practitioner in Yunnan Province, for the pathogenesis of PMO “deficiency of liver and kidney, Qi and blood disorder of muscles and bones”, and has been clinically proven to have excellent therapeutic effect for many years. The formula is composed of *Eucommia ulmoides* Oliv, *Psoralea corylifolia* L, *Cibcnium barometz* (L.) J. Sm, *Achyranthes bidentata* BI, *Chaenomeles speciosa* (Sweet) Nakai, *Morus alba* L, *Astragalus membranaceus* (Fisch.) Bge. var. mongholicus (Bge.) Hsiao, *Dipsacus asper* Wall, ex Henry, *Pueraria lobata* (Willd.) Ohwi,and *Parax notoginseng* (Burk.) F. H. Chen. It has the effect of tonifying the liver and kidneys, activating blood circulation and removing blood stasis, strengthening tendons and bones, and relaxing tendons and collaterals.

Based on this, the PMO rat model was replicated using bilateral ovarian denudation, and the model was judged by detecting bone mineral density, bone histopathologic changes, and serum indexes related to bone transformation in rats. Through the detection of gut microbiota, intestinal mucosal barrier, and intestinal inflammatory response-related indexes, we aimed to analyze the characteristics of gut microbiota in rats with PMO after the ZGSBG intervention to explore the correlation among the characteristic bacteria, the intestinal mucosal barrier, and the intestinal inflammatory response, and to investigate the role of the characteristic bacteria in the ZGSBG intervention in PMO. It provides a breakthrough for the study of the pharmacodynamic mechanisms of ZGSBG for the treatment of PMO and also provides an important reference for traditional Chinese medicine (TCM) to optimize its therapeutic effect on diseases by regulating gut microorganisms.

## 2 Materials and methods

### 2.1 Materials

#### 2.1.1 Animal

In total 30 female SD rats (10-weeks-old, 200 ± 20 g) were obtained from the Animal Experiment Center of Kunming Medical University (SCXK [Dian] K2020-0004; Kunming, China). The rats were maintained on a light-dark cycle of 12h at room temperature (23-25°C) and humidity (50%-70%) and were allowed unrestrained activity and free access to water and food. All animal experiments were reviewed and approved by the Ethical Review Committee for Animal Experiments of Yunnan University of Traditional Chinese Medicine (number: R-062021077).

#### 2.1.2 Reagents

The following ELISA kits were purchased from Jiangsu Meibiao Biotechnology Co., Ltd: secretory immunoglobulin A (SIgA) enzyme-linked immunosorbent assay (ELISA) kit (Lot. No: MB-1970A); tumor necrosis factor-α (TNF-α) ELISA kit (Lot. No: MB-1721A); interleukin-6 (IL-6) ELISA kit (Lot. No: MB-1731A); interleukin-1β (IL-1β) ELISA kit (Lot. No: MB-1588A); lipopolysaccharide (LPS) ELISA kit (Lot. No: MB-6601A); procollagen type I N-terminal propeptide (PINP) ELISA kit (Lot. No: MB-6809A); bone R-hydroxy glutamic acid protein (BGP) ELISA kit (Lot. No: MB-6809A); C-terminal telopeptide of type-I collagen (CTX-1) ELISA kit (Lot. No: MB-6809A); and tartrate-resistant acid phosphatase-5b (TRACP-5b) ELISA kit (Lot. No: MB-6809A).

#### 2.1.3 Medicine

Regarding the composition of ZGSBG, the suppliers and original product information of ZGSBG are shown in **Table 1**. The Chinese medicines in the table are all listed in the Chinese Pharmacopoeia (2020 edition), purchased from “Yixintang” Pharmacy in Kunming, Yunnan Province, and identified by the Department of Traditional Chinese Medicine Appraisal of Yunnan University of Traditional Chinese Medicine for their variety and quality.

**Table 1:** The suppliers and original product information of ZGSBG.

Chinese name	Latin name	Suppliers	Lot. no
Du zhong	Bark of *Eucommia ulmoides* Oliv	Yunnan Hongxiang Chinese Medicine technology Co., LTD	20220301
Bu guzhi	Ripe fruit of *Psoralea corylifolia* L.	Haozhou Yonggang Decoction piece factory Co., LTD	A220114
Jinmao gouji	Root of *Cibcnium barom etz* (L.) J. Sm	Yunnan Hongxiang Chinese Medicine technology Co., LTD	200301
Huai niuxi	Root of *Achyranthes bidentata* BI	Sichuan Boren Pharmaceutical Co., LTD	220601
Mu gua	Ripe fruit of *Chaenomeles speciosa* (Sweet) Nakai	Haozhou Yonggang Decoction piece factory Co., LTD	210105
Sang zhi	Shoot of *Morous alba.* L	Yunnan Hongxiang Chinese Medicine technology Co., LTD	20211001
Huang qi	Root of *Astragalus membranaceus* (Fisch.) Bge. var. *mongholicus* (Bge.) Hsiao	Yunnan Hongxiang Chinese Medicine technology Co., LTD	20201101
Xu duan	Root of *Dipsacus asper* Wall, ex Henry	Haozhou Yonggang Decoction piece factory Co., LTD	A220529
Ge gen	Root of *Pueraria lobata* (Willd.) Ohwi	Sichuan Guoqiang Traditional Chinese Medicine Decoction Pieces liability Co., LTD	22030106
San qi	Root of *Parax notoginseng* (Burk.) F. H. Chen	Yunnan Hongxiang Chinese Medicine technology Co., LTD	20190101

### 2.2 Methods

#### 2.2.1 Animal grouping

After 1 week of acclimation, the rats were randomly divided into three groups (n=6 per group): the sham surgery (Sham) group, the ovariectomized (OVX) group, and the ZGSBG group.

#### 2.2.2 Replication model

The PMO model was replicated using the bilateral ovarian denervation method. The rats were anesthetized using an injection of 10% chloral hydrate. After fixation, a 2-3cm longitudinal incision was made in the middle of the lumbar spine to expose the abdominal cavity on both sides. In the OVX and ZGSBG groups, the fallopian tubes were ligated on both sides and the ovarian tissue was removed, while in the Sham group, only a small piece of adipose tissue was removed from around the ovarian tissue. The surgical procedures and treatments were the same as in the OVX group. Penicillin was injected intramuscularly for 3 days to prevent wound infection (80000 units/each), and the rats were kept in a single cage for 1 week to avoid any rupturing and bleeding of the surgical wounds caused by biting among the rats.

#### 2.2.3 Drug preparation and administration

The total amount of raw drugs administered in the ZGSBG group was 185 g. According to the conversion ratio of equivalent dose for body surface area in the “Methodology of Pharmacological Research of Traditional Chinese Medicines” (Li, 2006), the clinical equivalent dose was taken as the therapeutic amount, and the daily dosage of ZGSBG for each rat was 185×0.018 = 3.33 g/kg. The three-time filtrate was concentrated into a thick extract by decocting with water and placed in the refrigerator at -20°C for spare use.

Regarding the pharmacological interventions, after successful modeling, the ZGSBG group was gavaged with an aqueous decoction of ZGSBG of 2 mL/d, once a day, for 8 weeks, and the Sham and OVX groups were gavaged with isotonic sterile distilled water.

#### 2.2.4 High-performance liquid chromatography-tandem mass spectrometry of ZGSBG

The chemical components of the ZGSBG extracts were confirmed by fingerprinting analysis. The samples were identified using a Vanquish UHPLC system (Thermo Fisher, Germany) coupled with an Orbitrap Q ExactiveTMHF-X mass spectrometer (Thermo Fisher, Germany) in Novogene Co., Ltd. (Beijing, China). The samples were processed on a Hypesil Gold column (100×2.1 mm, 1.9 μm) using a 12-min linear gradient at a flow rate of 0.2mL/min. The Q ExactiveTM HF-X mass spectrometer was operated in positive/negative polarity mode with a spray voltage of 3.5 kV, capillary temperature of 320°C, sheath gas flow rate of 35 psi, aux gas flow rate of 10 L/min, S-lens RF level of 60, and aux gas heater temperature of 350°C. See **Table 2** for details.

**Table 2:** Mobile phase condition of chormatographic separation.

Times (min)	Mobile phase A (%)	Mobile phase B (%)
0	98	2
1.5	98	2
3	15	85
10	0	100
10.1	98	2
11	98	2
12	98	2

Positive mode, Mobile phase A: 0.1% formic acid; Mobile phase B: methanol. Negative mode, Mobile phase A: 5 mM ammonium acetate, pH=9.0; Mobile phase B: methanol.

#### 2.2.5 Micro computed tomography technique evaluated the density of femur tissue

A NEMO micro computed tomography (CT) system from PINGSENG Healthcare Inc. was used to scan the femur of rats fixed to the micro CT carrier table. The analysis of trabecular bone mineral density (Tb. BMD), trabecular number (Tb. N), trabecular thickness (Tb. Th), and trabecular separation (Tb. Sp) was performed using the on-board software.

#### 2.2.6 Hematoxylin-eosin staining observed the structural changes of femur and colon tissues

Rats in each group were anesthetized by intraperitoneal injection of 10% chloral hydrate. Under aseptic conditions, the connective tissue was removed from the femoral and colon tissues, fixed in 4% paraformaldehyde solution, dehydrated with gradient ethanol, made transparent with xylene, embedded in conventional paraffin, sliced, and stained by hematoxylin-eosin (HE). The structural changes of the femurs and colons were observed under an optical microscope.

#### 2.2.7 Masson staining observed the collagen deposition of femur tissue

The femur tissue was fixed, dehydrated, made transparent, paraffin-embedded, and sliced. The slices were soaked in a ponceau solution, a weak acid solution, a phosphomolybdic acid solution, and an aniline blue solution respectively. They were then quickly dehydrated with high-concentration alcohol. After soaking in anhydrous ethanol, the xylene is transparent and neutral gum sealed. The collagen deposition in the tissues was observed under an optical microscope.

#### 2.2.8 ELISA detected the levels of BGP, PINP, CTX-1, and TRACP-5b in serum

Blood was collected from the abdominal aortas in each group, and blood was collected using blood collection vessels without anticoagulant. After standing for 30 min, the samples were centrifuged at 3500 rpm at 4°C for 15 min, and the supernatant was absorbed with a pipette and loaded into a sterilized centrifuge tube. The plate layout was set, the sample added, the enzyme added, and then incubated. The plate was then washed, the color developed, and the reaction was stopped. The samples were then tested on the machine according to the methods provided in the ELISA kits.

#### 2.2.9 ELISA detected the levels of SIgA, IL-6, TNF-α, IL-1β and LPS in colon tissue

The collected colon tissue samples were ground, and the supernatant was taken for plate layout, sample addition, enzyme addition, incubation, plate washing, color rendering, reaction termination, and machine detection according to the methods provided in the ELISA kits.

#### 2.2.10 Immunofluorescence analyzed the expressions of claudin-1, occludin and zonula occludens-1 in colon tissue

The collected colon tissue was successively subjected to paraffin section dewaxing, antigen repair, serum sealing, antibody incubation, DAPI restaining, quenching of tissue autofluorescence, sealing, and other steps, and the collected images were observed under a fluorescence microscope.

#### 2.2.11 16S rRNA gene high-throughput sequencing detected the gut microbiota

Fresh fecal samples were collected on the day of sampling. The samples from each rat were placed individually in sterile EP tubes, labeled, and stored in a refrigerator at -80°C for 16S rRNA gene subsequent high-throughput sequencing. 16S rRNA gene high-throughput sequencing included microbiome total DNA extraction and amplification, PCR amplification, amplification product recovery and purification, library preparation and library inspection, computer sequencing, and other steps. The sequencing was completed by Shanghai Baiqu Biomedical Technology Co., LTD.

### 2.3 Data statistics

The experimental data were statistically analyzed and plotted using IBM SPSS Statistics 22 and GraphPad Prism 9. The data were represented by mean ± standard deviation. One-way ANOVA and t-test were selected as the testing methods. *P*<0.05 was statistically significant and *P*>0.05 was not statistically significant.

## 3 Results

### 3.1 Components analysis of ZGSBG

**Figure 1:**
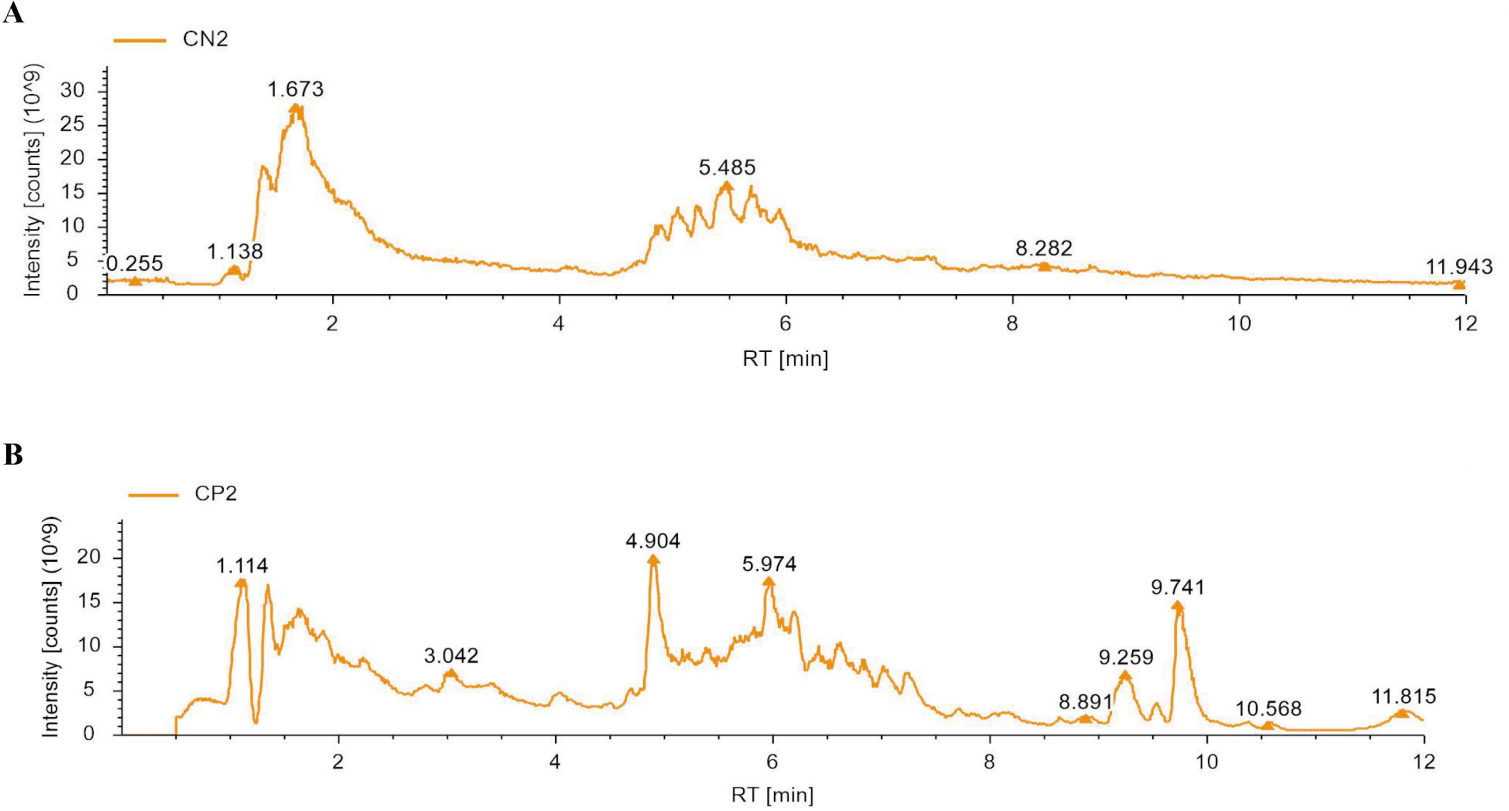
Mass spectrum chromatograms of ZGSBG. **(A)** Negative mode. **(B)** Positive mode.

To identify the major chemical components, ZGSBG was analyzed using high-performance liquid chromatography-tandem mass spectrometry (HPLC-MS/MS), combining the high-quality mnzCloud database built by standard products with the mzVaut and MasList databases to match and identify molecular characteristic peaks. The total positive and negative ion chromatograms of ZGSBG demonstrated the chemical composition of all the compounds (**Figure 1**). A total of 133 active compounds were confirmed in ZGSBG. These active components were ranked according to the number of active components in ZGSBG. Active components of ZGSBG mainly included flavonoids, amino acids, purine, phenylpropanoids, and vitamins, for example, formononetin, L-Pyroglutamic acid, L-Phenylalanine, L-Tyrosine, guanine, isoflavones, puerarin, nicotinamide, and D-(-)-Quinic acid (**Table 3**).

**Table 3:** Identification of components of ZGSBG.

Name	Formula	Molecular Weight	RT(min)	Peak area(neg)
DL-Malic acid	C_4_H_6_O_5_	134.02152	1.551	2.92E+11
Citric acid	C_6_H_8_O_7_	192.02705	1.737	1.83E+11
Oleamide	C_18_H_35_NO	281.27161	9.763	1.41E+11
4-Oxoproline	C_5_H_7_NO_3_	129.04268	2.267	33333262631
L-Pyroglutamic acid	C_5_H_7_NO_3_	129.04275	2.169	32730159675
L-Phenylalanine	C_9_H_11_NO_2_	165.07906	4.921	22990645184
Pipecolic acid	C_6_H_11_NO_2_	129.07912	1.694	18691220987
Guanine	C_5_H_5_N_5_O	151.04941	3.456	15296197314
L-Tyrosine	C_9_H_11_NO_3_	181.07401	2.326	13807556236
Adenosine	C_10_H_13_N_5_O_4_	267.09664	3.031	13588814800
D-(-)-Quinic acid	C_7_H_12_O_6_	192.06358	5.246	10813124220
D-(+)-Mannose	C_6_H_12_O_6_	180.06346	1.342	10592000228
Azelaic acid	C_9_H_16_O_4_	188.10503	5.837	10340180288
4-Hydroxybenzaldehyde	C_7_H_6_O_2_	122.03685	5.521	8049228289
2-Isopropylmalic acid	C_7_H_12_O_5_	176.06858	5.469	7853837585
Puerarin	C_21_H_20_O_9_	416.11046	5.276	7594625756
Guanosine	C_10_H_13_N_5_O_5_	283.09151	3.477	6941043330
α,α-Trehalose	C_12_H_22_O_11_	342.11595	1.429	6728520364
Nicotinamide	C_6_H_6_N_2_O	122.04809	2.026	5248148956
Formononetin	C_16_H_12_O_4_	268.07351	6.233	5145339438

### 3.2 Effects of ZGSBG on femoral density, structure and bone metabolism in PMO rats

#### 3.2.1 ZGSBG enhanced the bone density in PMO rats

**Figure 2:**
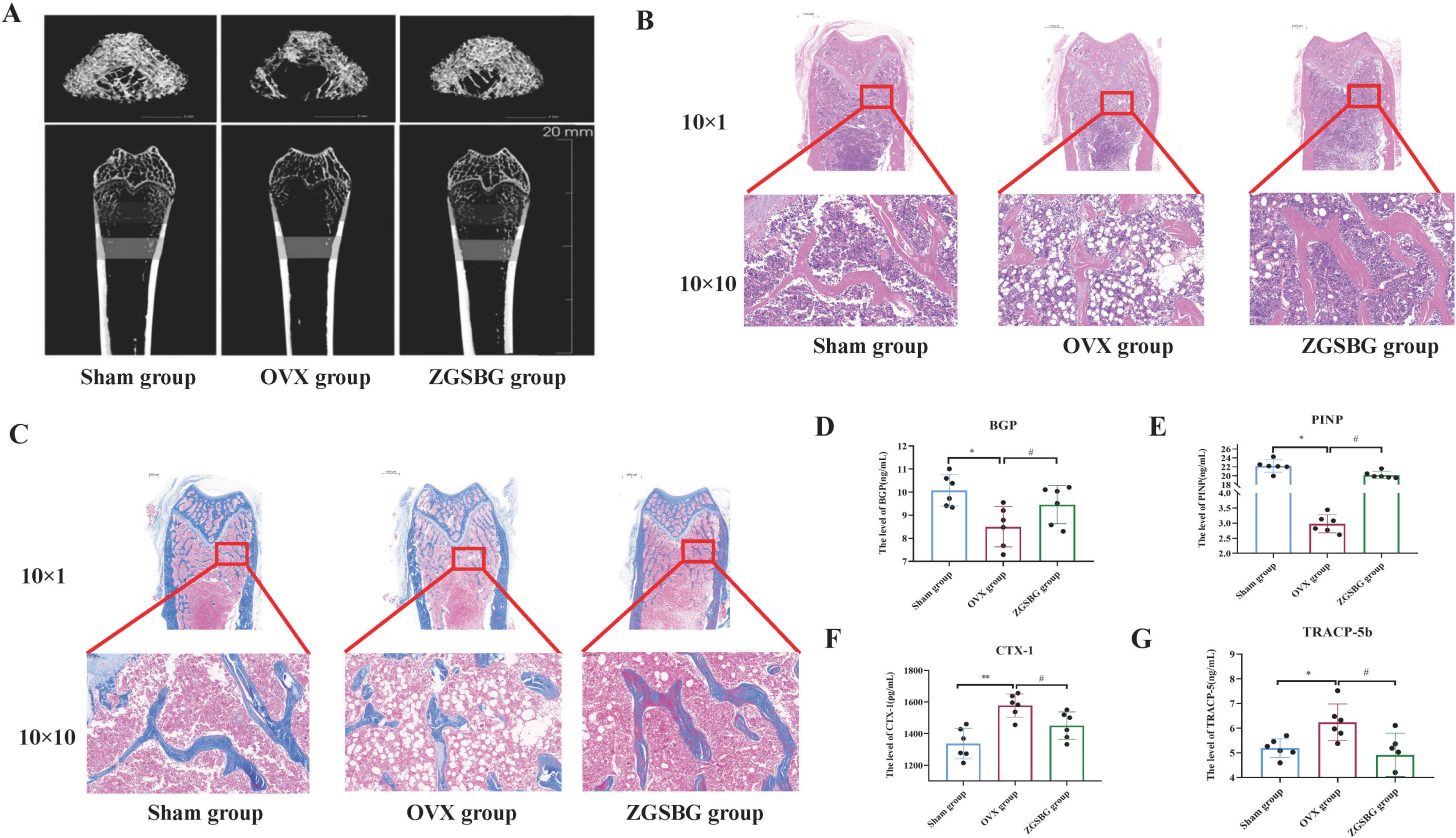
Effects of ZGSBG on femur bone density, structure, and bone metabolism in rats with PMO. **(A)** Observation of the structural changes of rat femur using the micro CT technique. **(B)** HE staining to observe the structural changes in the femur (upper column: 10×1; lower column: 10×10). **(C)** Masson staining to observe the structural changes in the femur (upper column: 10×1; lower column: 10×10). **(D)** BGP level in serum. **(E)** PINP level in serum. **(F)** CTX-1 level in serum. **(G)** TRACP-5b level in serum. ^*^ *P*<0.05 and **P<0.01, compared with the Sham group; ^#^ *P*<0.05, compared with the OVX group. Sham group, sham surgery group; OVX group, ovariectomized group; ZGSBG group, Zhuanggu Shubi ointment group.

Compared with the Sham group, Tb.BV/TV, Tb.BMD, Tb.Th, and Tb.N in the OVX group significantly decreased (*P*<0.05; *P*<0.05; *P*<0.05; *P*<0.05), however, Tb.Sp showed an increasing trend (*P*>0.05). Compared with the OVX group, Tb.BMD, Tb.BV/TV, Tb.Th, and Tb.N in the ZGSBG group increased (*P*>0.05; *P*<0.05; *P*<0.05; *P*<0.05) but Tb.Sp showed a decreasing trend (*P*>0.05) (**Table 4**, **Figure 2A**). Thus, it can be shown that ZGSBG enhanced the bone density of femur tissue in PMO rats.

**Table 4:** Effects of ZGSBG on bone mineral density in PMO rats (n=3, x± s).

Group	Tb. BV/TV (%)	Tb. BMD (g/cm^3)	Tb. Th (mm)	Tb. N (mm^-1)	Tb. Sp (mm)
Sham group	0.28±0.02	1.97±0.03	0.13±0.01	2.04 ± 0.09	0.37 ± 0.02
OVX group	0.10 ± 0.05^*^	1.92±0.01^*^	0.11±0.00^*^	1.02 ± 0.39^*^	0.96 ± 0.38
ZGSBG group	0.26 ± 0.03^#^	1.93±0.01	0.12±0.00^#^	1.98 ± 0.39^#^	0.39 ± 0.10

(1) Compared with Sham group, ^*^ *P* < 0.05; (2) Compared with OVX group, ^#^ *P* < 0.05. Sham group: Sham surgery group; OVX group: Ovariectomized group; ZGSBG group: Zhuanggu Shubi Ointment.

#### 3.2.2 ZGSBG improved femoral structural injury in PMO rats

HE staining showed that the bone trabeculae in the Sham group were thick, neatly arranged, and had good continuity. Compared with the Sham group, the OVX group had more bone trabecular junction breakpoints, thinner and sparser bone trabecular distribution, and more fat cells in the bone marrow cavity. After the ZGSBG intervention, the above conditions were improved (**Figure 2B**).

Masson staining indicated that the collagen fibers of the femurs in the Sham group were dyed dark blue and the collagen fibers were arranged neatly. Compared with the Sham group, the collagen fiber staining of the OVX group was light blue, the collagen fiber junction breakpoints increased, and the collagen fiber distribution was sparse. After ZGSBG intervention, the above conditions were improved (**Figure 2C**). These results showed that ZGSBG improved femoral structural injury in PMO rats.

#### 3.2.3 ZGSBG regulated the bone metabolic in PMO rats

Compared with the Sham group, the levels of BGP and PINP in the OVX group significantly decreased (*P*<0.05; *P*<0.05) (**Figures 2D, E**) and CTX-1 and TRACP-5b significantly increased (*P*<0.05; *P*<0.05) (**Figures 2F, G**). Compared with the OVX group, BGP and PINP levels in the ZGSBG group were significantly elevated (*P*<0.05; *P*<0.05), and CTX-1 and TRACP-5b were significantly decreased (*P*<0.05; *P*<0.05). The above results indicated that ZGSBG promoted bone formation and inhibited bone resorption in PMO rats.

### 3.3 Effects of ZGSBG on the intestinal mucosal barrier in PMO rats

#### 3.3.1 ZGSBG upregulated the expression of the tight junction protein in PMO rats

**Figure 3:**
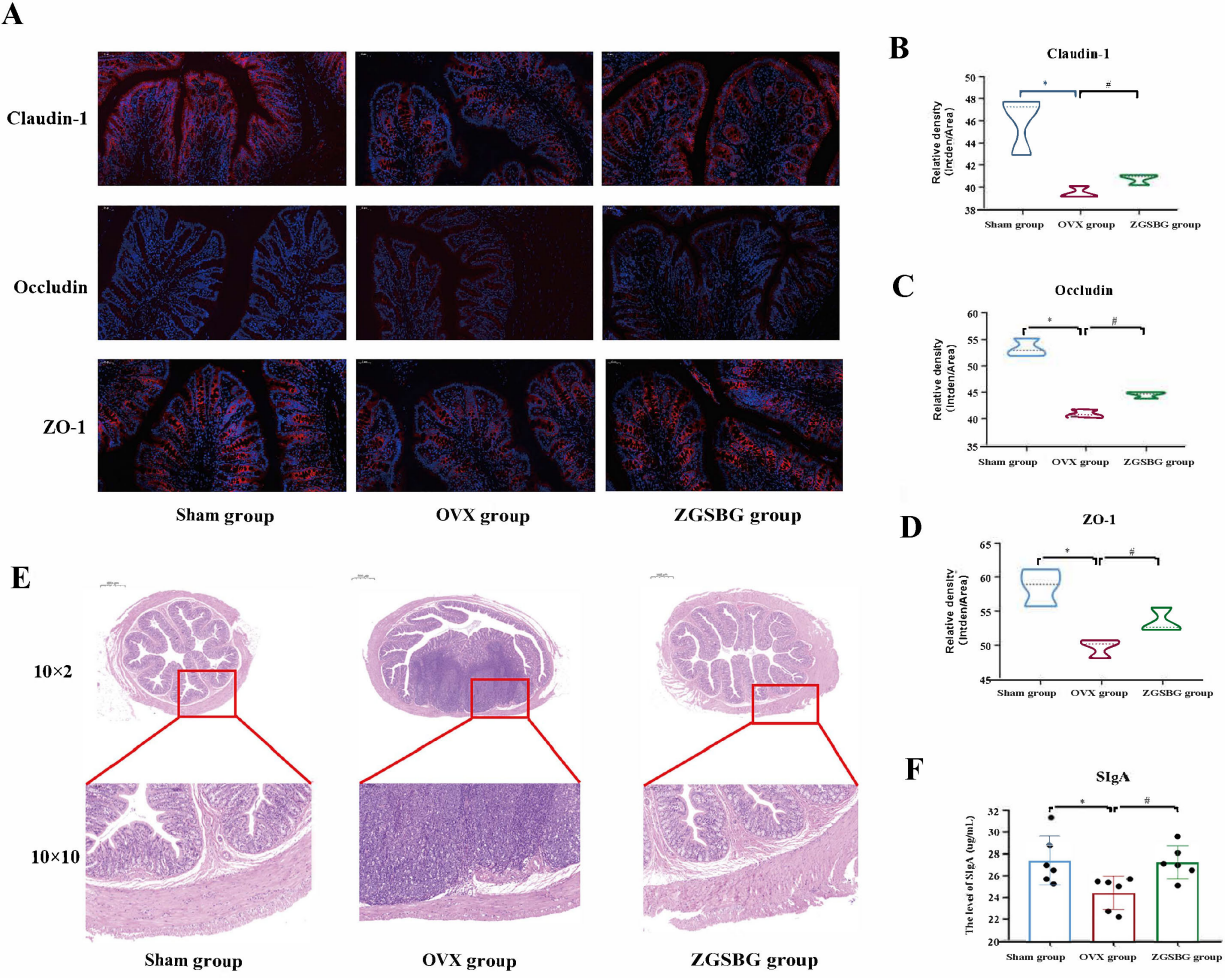
Effects of ZGSBG on the intestinal mucosal barrier function of PMO rats. **(A)** Immunofluorescence detection of claudin-1, occludin, ZO-1 in colonic tissue. **(B)** Gray scale values of claudin-1. **(C)** Gray-scale values of occludin. **(D)** Gray scale values of ZO-1. **(E)** HE staining to observe the structural changes in colonic tissue (10×1 in the upper column; 10×10 in the lower column). **(F)** SIgA level in colonic tissue. ^*^ *P*<0.05, compared with the Sham group; ^#^ *P*<0.05, compared with the OVX group. Sham group, sham surgery group; OVX group, ovariectomized group; ZGSBG group, Zhuanggu Shubi ointment group.

The relative expressions of claudin-1, occludin, and zonula occludens-1 (ZO-1) in the OVX group were significantly lower compared to the Sham group (*P*<0.05; *P*<0.05; *P*<0.05). After the ZGSBG intervention, the above indexes significantly increased (*P*<0.05; *P*<0.05; *P*<0.05) (**Figures 3A–D**), demonstrating that ZGSBG upregulated the expression of the tight junction protein in PMO rats.

#### 3.3.2 ZGSBG improved the colonic tissue structure damage in PMO rats

Compared with the Sham group, the rats in the OVX group presented with shallow intestinal crypt distortion, edema of the submucosa, and a certain degree of inflammatory cell infiltration in the basal layer. The above conditions were improved after the ZGSBG intervention (**Figure 3E**). Thus, this shows that ZGSBG improved colonic tissue structure damage in PMO rats.

#### 3.3.3 ZGSBG elevated the level of SIgA in PMO rats

Compared with the Sham group, the level of SIgA in the colonic tissue of the rats in the OVX group was significantly lower (*P*<0.05), and compared with the OVX group, the SIgA level in the ZGSBG group was significantly higher (*P*<0.05) (**Figure 3F**), which showed that ZGSBG elevated the level of SIgA in PMO rats.

### 3.4 Effect of ZGSBG on the intestinal inflammatory response in PMO rats

**Figure 4:**
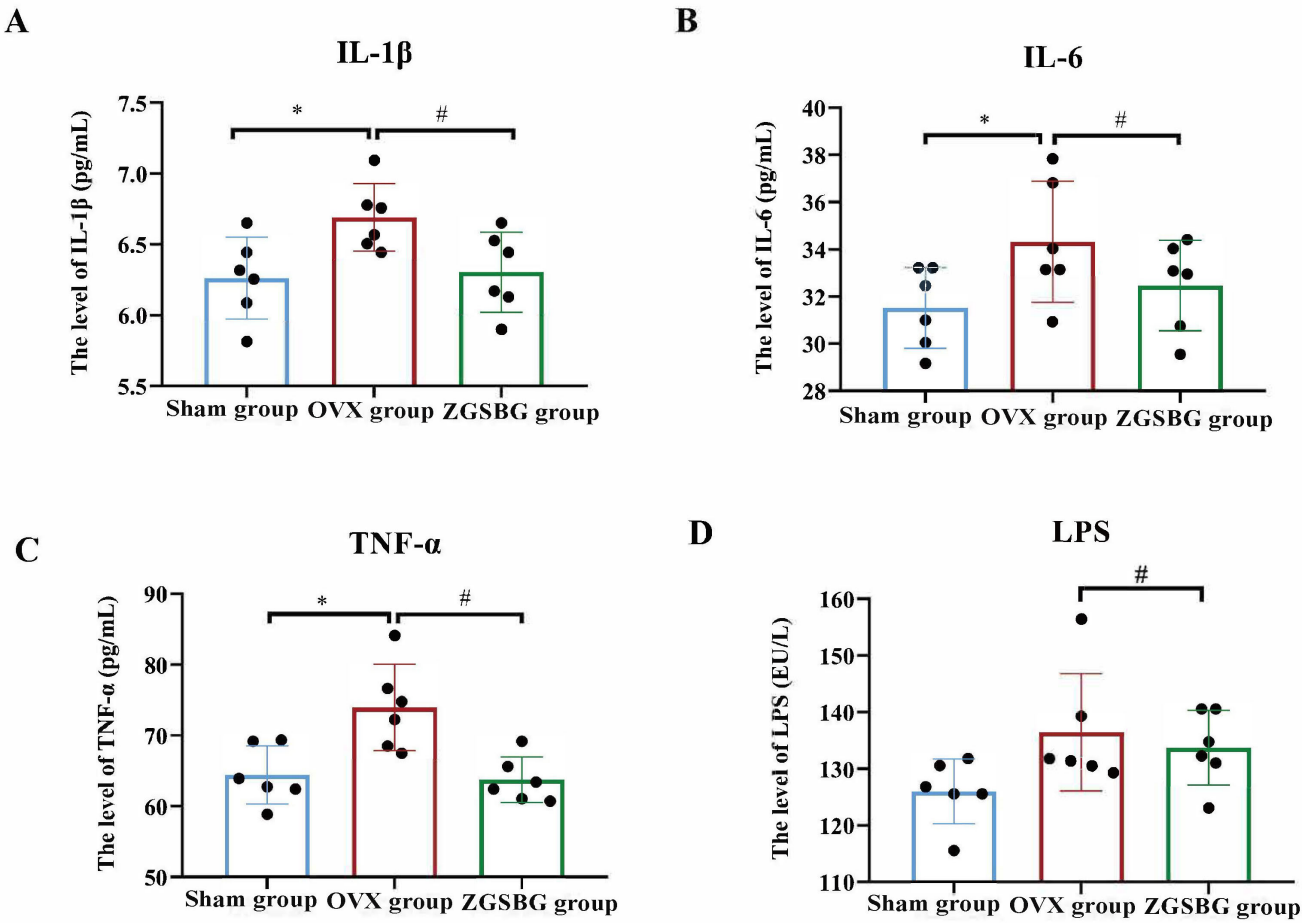
Effect of ZGSBG on the intestinal inflammatory response in rats with PMO. **(A)** IL-1β level in colon tissue. **(B)** IL-6 level in colon tissue. **(C)** TNF-α level in colon tissue. **(D)** LPS level in colon tissue. ^*^ *P*<0.05, compared with the Sham group; ^#^ *P*<0.05, compared with the OVX group. Sham group, sham surgery group; OVX group, ovariectomized group; ZGSBG group, Zhuanggu Shubi ointment group.

Compared with the Sham group, the levels of TNF-α, IL-6, and IL-1β in the colonic tissues of the rats in the OVX group were greatly increased (*P*<0.05; *P*<0.05; *P*<0.05), and LPS showed an increasing trend (*P*>0.05). Compared with the OVX group, TNF-α, IL-1β, IL-6, and LPS in the ZGSBG group decreased significantly (*P*<0.05; *P*<0.05; *P*<0.05; *P*<0.05; *P*<0.05) (**Figure 4**), demonstrating that ZGSBG alleviated the intestinal inflammatory response of PMO rats.

### 3.5 Effect of ZGSBG on the gut microbiota in PMO rats

#### 3.5.1 Quality assessment of sequencing data

**Figure 5:**
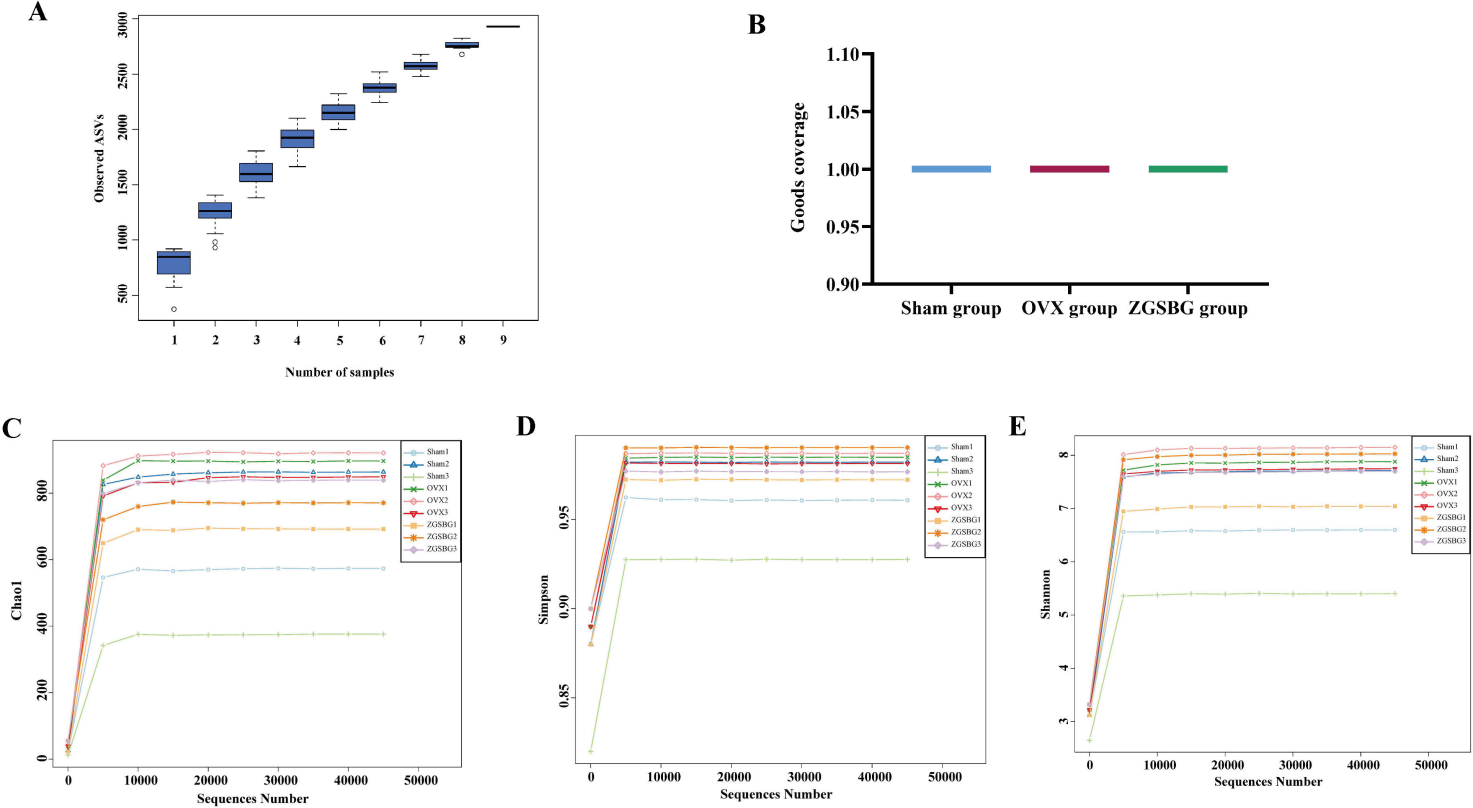
Quality assessment of sequencing data analysis of gut microbiota. **(A)** Species accumulation curves. **(B)** Good’s coverage index. **(C)** Chao1 dilution curve. **(D)** Simpson dilution curve. **(E)** Shannon dilution curve. Sham group, sham surgery group; OVX group, ovariectomized group; ZGSBG group, Zhuanggu Shubi ointment group.

As the sample size increased, the total number of OTUs barely increased with the addition of new samples, suggesting that the sampling in this study was sufficiently adequate to meet the needs of the study (**Figure 5A**). Good’s coverage index of the samples within the same group was above 99.5%, indicating that the coverage of the samples within the group was good and that there were no samples with large outliers (**Figure 5B**). The number of sequenced sequences in all samples was greater than 50,000, and the maximum sequencing depth was reached when the number of sequences reached 5,000 (**Figures 5C–E**), suggesting that the two groups of samples were sequenced in sufficient depth to meet the requirements of subsequent studies. In brief, it was demonstrated that the experimental data met the needs of the experimental design and downstream analysis.

#### 3.5.2 ZGSBG adjusted the diversity of gut microbiota in PMO rats

**Figure 6:**
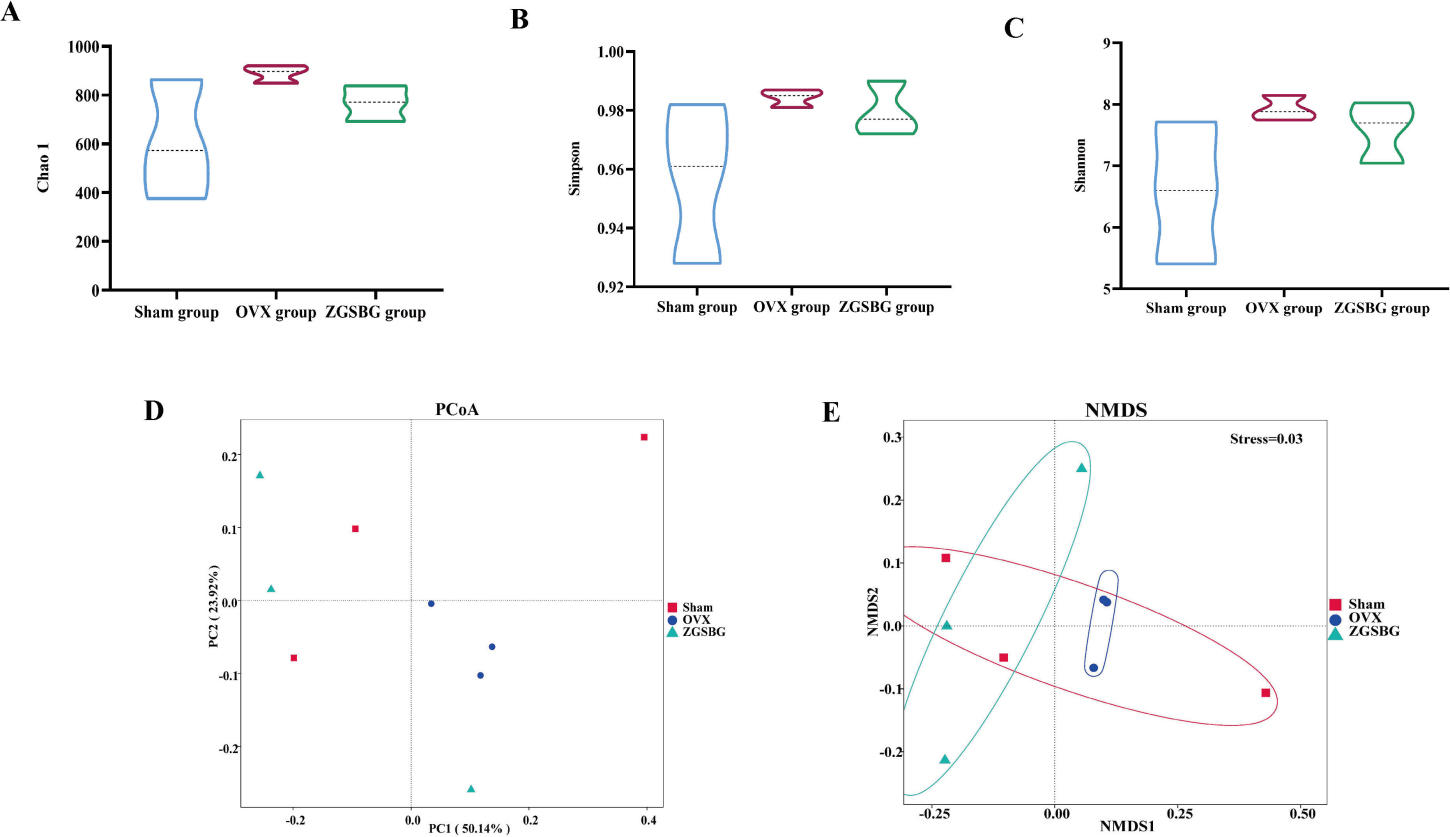
Effects of ZGSBG on the diversity and community structure of gut microbiota in rats with PMO. **(A)** Chao1 index. **(B)** Simpson index. **(C)** Shannon index. **(D)** PCoA analysis. **(E)** NMDS analysis. Sham group, sham surgery group; OVX group, ovariectomized group; ZGSBG group, Zhuanggu Shubi ointment group.

Compared with the Sham group, the Chao 1, Simpson, and Shannon indexes in the OVX group presented increasing trends (*P>*0.05; *P>*0.05; *P>*0.05). Compared with the OVX group, the above indexes in the ZGSBG group presented decreasing trends (*P>*0.05; *P>*0.05; *P>*0.05) (**Figures 6A–C**). Principal coordinates analysis (PCoA) is a non-binding data dimensionality reduction method used to study the similarities or differences in sample community composition. In this study, the community similarity and difference between the OVX group and the ZGSBG group were demonstrated by PCoA using two-dimensional visual scatter plots. In PCo1 and PCo2, the weighted UniFrac algorithm was used to calculate the distance (contribution value) between samples of the two groups to reflect the degree of aggregation and dispersion of the sample communities. In the PCoA (**Figure 6D**), the contribution rate of horizontal PCo1 was 50.14%, and that of vertical PCo2 was 23.92%. Non-metric multidimensional scaling (NMDS) analysis showed that the three groups of gut microbiota communities had different structural distribution characteristics with a stress value of 0.03, indicating that the grouping was reasonable (**Figure 6E**). Altogether, ZGSBG adjusted the diversity of gut microbiota in PMO rats.

#### 3.5.3 ZGSBG reshaped the dominant bacteria in PMO rats

**Figure 7:**
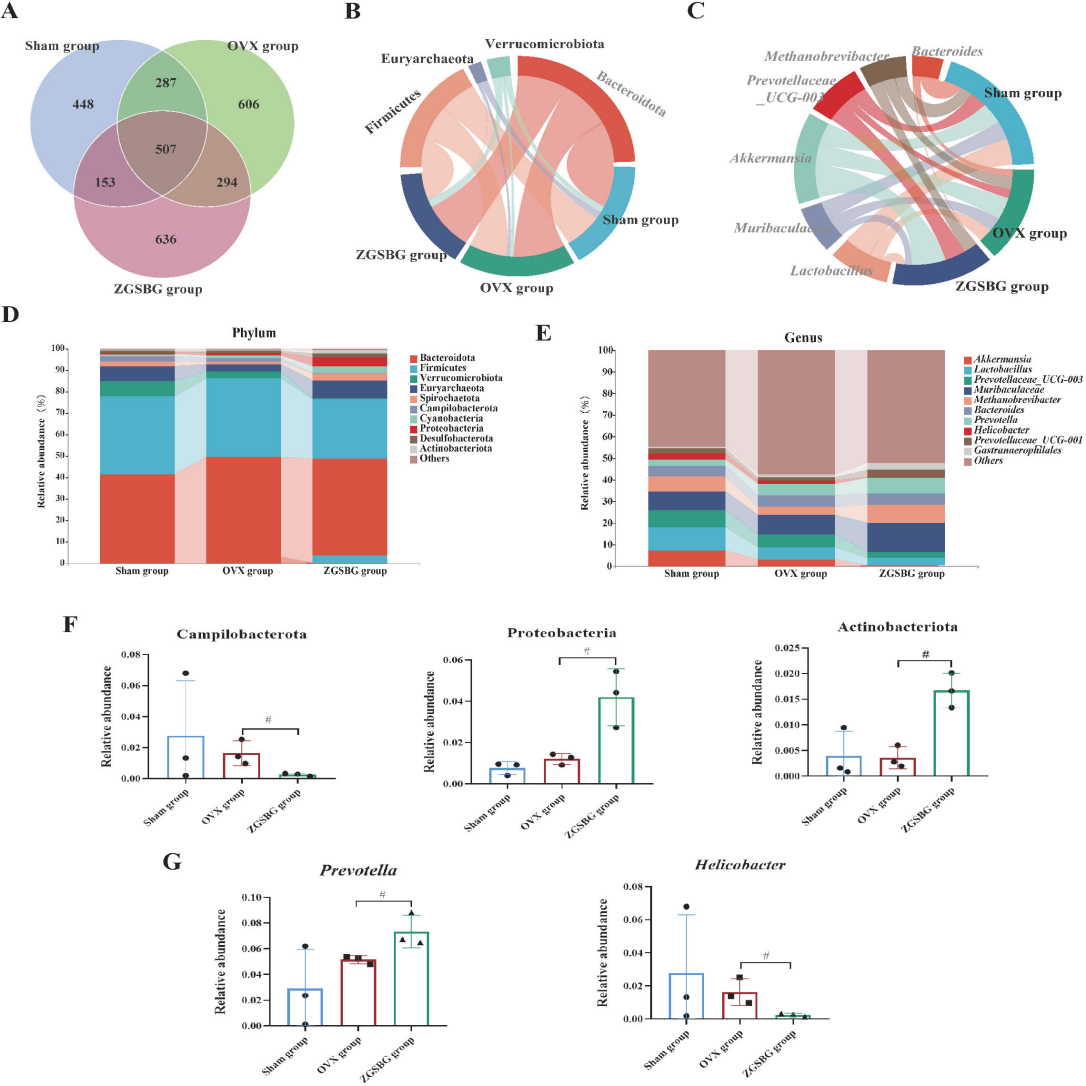
Effect of ZGSBG on the composition of gut microbiota in rats with PMO. **(A)** Venn diagram. **(B)** Relative abundance diagram of the phylum level. **(C)** Chord plot at the phylum level. **(D)** Relative abundance diagram of the genus level. **(E)** Chord plot at the genus level. **(F)** Dominant phyla with significant variation. **(G)** Dominant genera with significant variation. ^#^ *P*<0.05, compared with the OVX group. Sham group, sham surgery group; OVX group, ovariectomized group; ZGSBG group, Zhuanggu Shubi ointment group.

The Sham group had 1395 OTUs and 448 OTUs exclusively. The OVX group had 1,694 OTUs and 606 OTUs exclusively. The ZGSBG group had 1,590 OTUs and 636 OTUs exclusively. The total number of OTUs in the three groups was 507 (**Figure 7A**).

At the phylum level, Bacteroidetes, Firmicutes, Verrucomicrobiota, Euryarchaeota, and Spirochaetota were the predominant bacteria in the three groups (**Figure 7B**). On the basis of the top 10 species in relative abundance, we applied a chord diagram to summarize the dominant bacteria with abundances greater than 1% (**Figure 7D**). After statistical analysis, the relative abundance of Proteobacteria and Actinobacteriota in the ZGSBG group was significantly higher than the OVX group (*P*<0.05; *P*<0.05) and Campilobacterota was notably lower (*P*<0.05) (**Figure 7F**).

At the genus level, *Akkermansia*, *Lactobacillae*, *Prevotellaceae_UCG-003*, *Muribaculaceae* and *Bacteroides* were the predominant bacteria in the three groups (**Figure 7C**). On the basis of the top 10 species in relative abundance, we applied a chord diagram to summarize the dominant bacteria with abundances greater than 1% (**Figure 7E**). After statistical analysis, the relative abundance of *Prevotella* in the ZGSBG group was significantly higher than the OVX group (*P*<0.05) and *Helicobacter* was significantly lower (*P*<0.05) (**Figure 7G**).

Thus, the composition of the dominant bacteria in the rats changed at the phylum and genus levels after ZGSBG intervention, with Proteobacteria, Actinobacteriota, Campilobacterota, *Prevotella*, and *Helicobacter* changing significantly.

#### 3.5.4 ZGSBG enriched the characteristic bacteria in PMO rats

**Figure 8:**
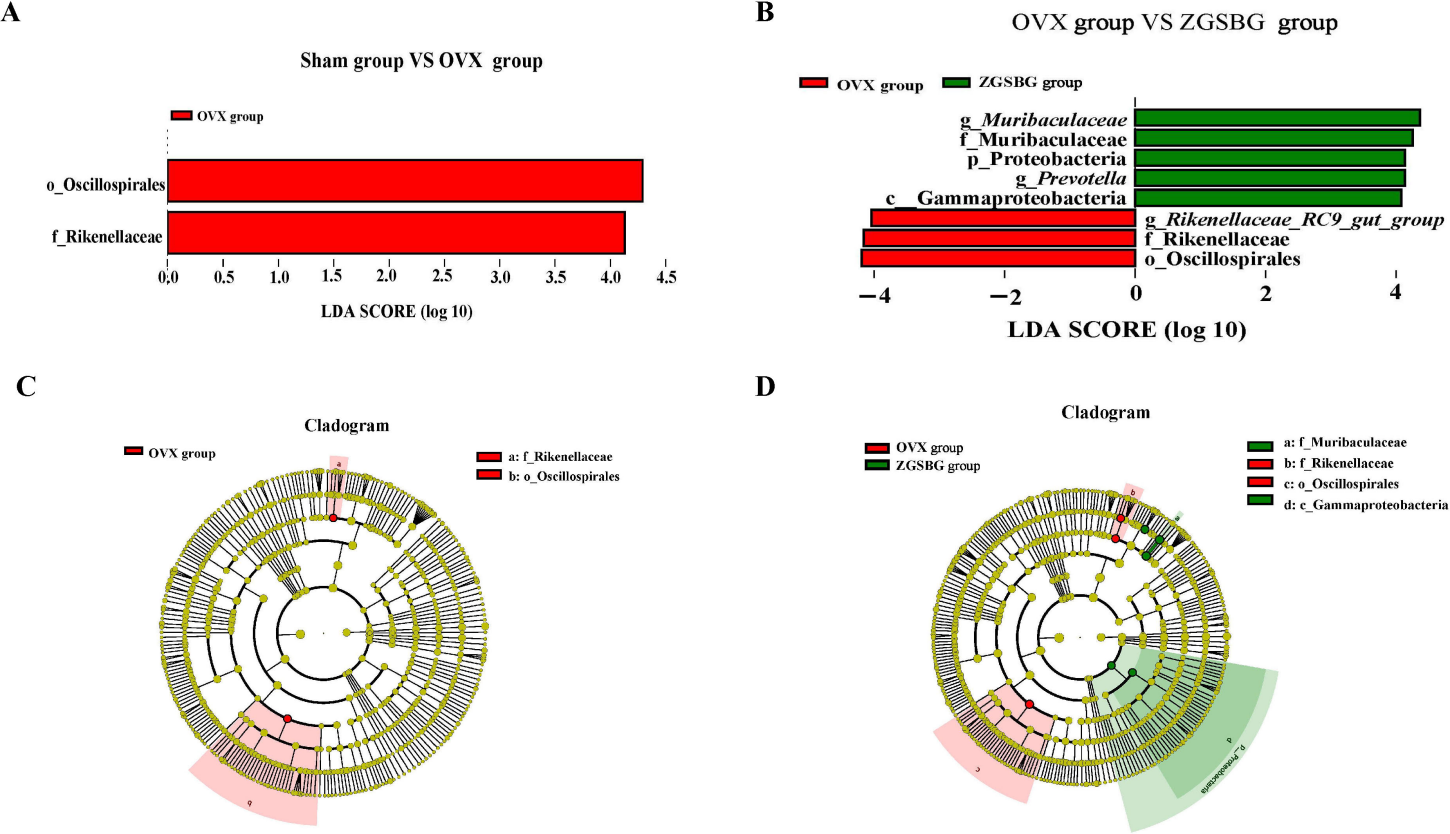
Effect of ZGSBG on gut characteristic bacteria of PMO rats. **(A)** LDA plot (Sham group vs. OVX group). **(B)** LDA plot (OVX group vs. ZGSBG group). **(C)** Cladogram plot (Sham group vs. OVX group). **(D)** Cladogram plot (OVX group vs. ZGSBG group). Sham group, sham surgery group; OVX group, ovariectomized group; ZGSBG group, Zhuanggu Shubi ointment group.

Linear discriminant analysis Effect Size (LEfSe) analysis mainly conducts linear discriminant analysis (LDA) on samples according to different grouping conditions based on taxonomic composition and identifies communities or species that have significant differential effects on sample classification. There were two differential taxa between the Sham group and the OVX group, namely Oscillospirales and Rikenellaceae (**Figure 8A**). In contrast, there were seven differential taxa between the OVX group and the ZGSBG group (**Figure 8B**), where the major bacteria in the ZGSBG group were *Muribaculaceae*, Muribaculaaceae, Proteobacteria, *Prevotella*, and Gammaproteobacteria. The results showed that there were significant differences in characteristic bacteria among the three groups. **Figures 8C, D** further show that the intestinal microbial structure of the Sham group, OVX group, and ZGSBG group was different.

#### 3.5.5 ZGSBG affected the function of gut microbiota in PMO rats

**Figure 9:**
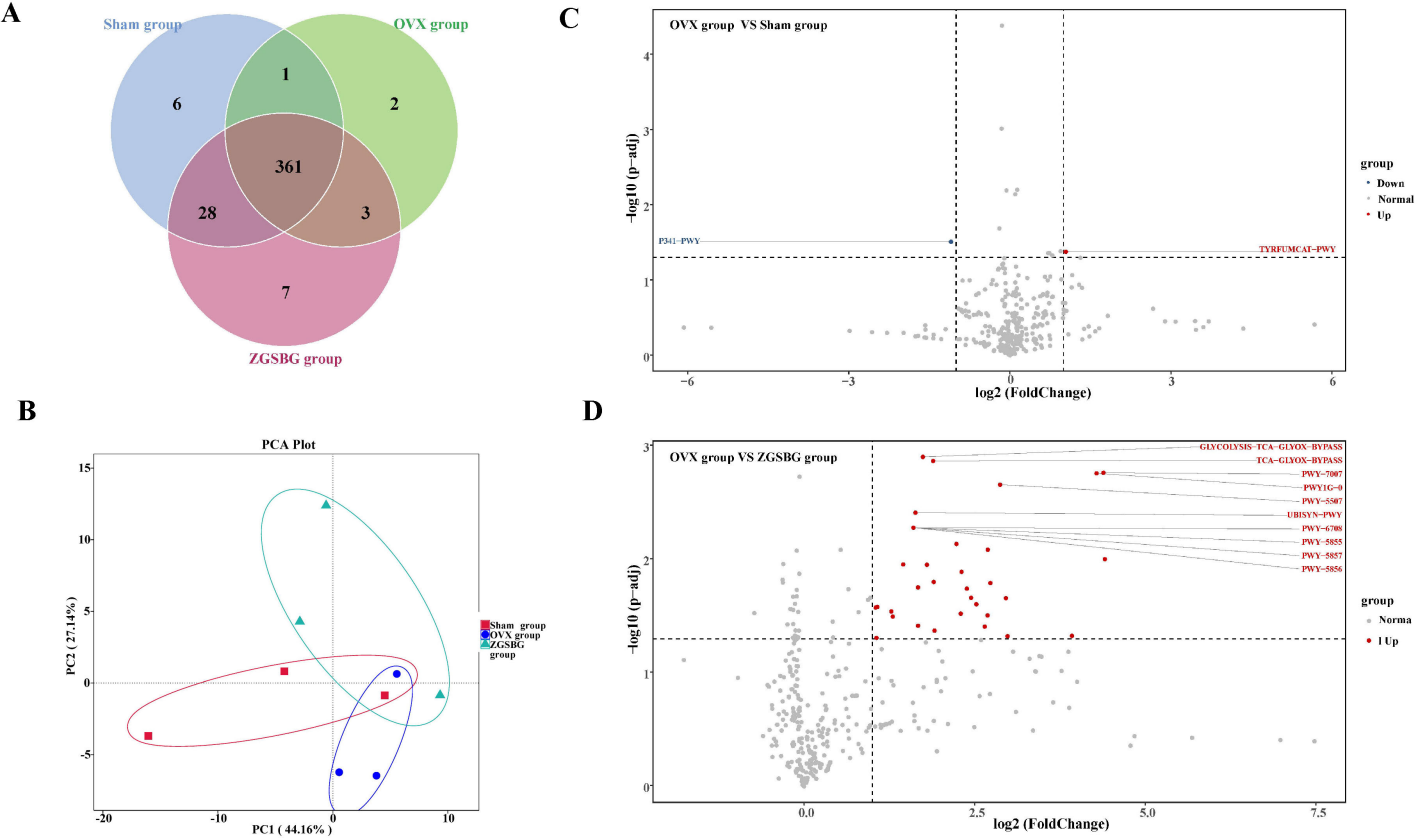
Effect of ZGSBG on the function of gut microbiota in PMO rats. **(A)** Venn diagram. **(B)** PCA plot. **(C)** Differential functional pathway volcano plot (OVX group vs. Sham group). **(D)** Differential functional pathway volcano plot (OVX group vs. ZGSBG group). Sham group, sham surgery group; OVX group, ovariectomized group; ZGSBG group, Zhuanggu Shubi ointment group.

There were a total of 396 functional pathways in the Sham group, 6 of which were unique, as shown in **Figure 9A**. The OVX group has a total of 367 unique functional pathways and the ZGSBG group has a total of 399, including 7 unique ones. There were 361 functional pathways in the three groups. It can be seen that ZGSBG promoted the enrichment of gut microbiota functional pathways in PMO rats. In the principal component analysis (PCA) (**Figure 9B**), the contribution rate of abscissa PC1 was 44.16%, and that of ordinate PC2 was 27.14%. The three groups showed a certain degree of dispersion, indicating that the functional structure of the gut microbiota in the PMO rats changed after the ZGSBG intervention. Subsequently, the data of two groups i.e., the OVX group vs. Sham group and the OVX group vs. ZGSBG group, were mapped to the KEGG database using Picrust2 software to predict the functional pathways of the microflora (**Figures 9C, D**). The analysis of the OVX group vs Sham group included a downregulated functional pathway (P341-PWY) and an upregulated functional pathway (TYRFUMCAT-PWY) with significant statistical differences. The analysis of the OVX group vs ZGSBG group included 31 upregulated functional pathways. Furthermore, statistically significant differences were found in the Glycolysis-TCA-GLyox-bypass, TCA-GLyox-bypass, and PWY-7007 pathways. Thus, the above metabolic pathways might be the main ways that ZGSBG caused the changes in the gut microbiota in PMO rats.

### 3.6 Correlation analysis

**Figure 10:**
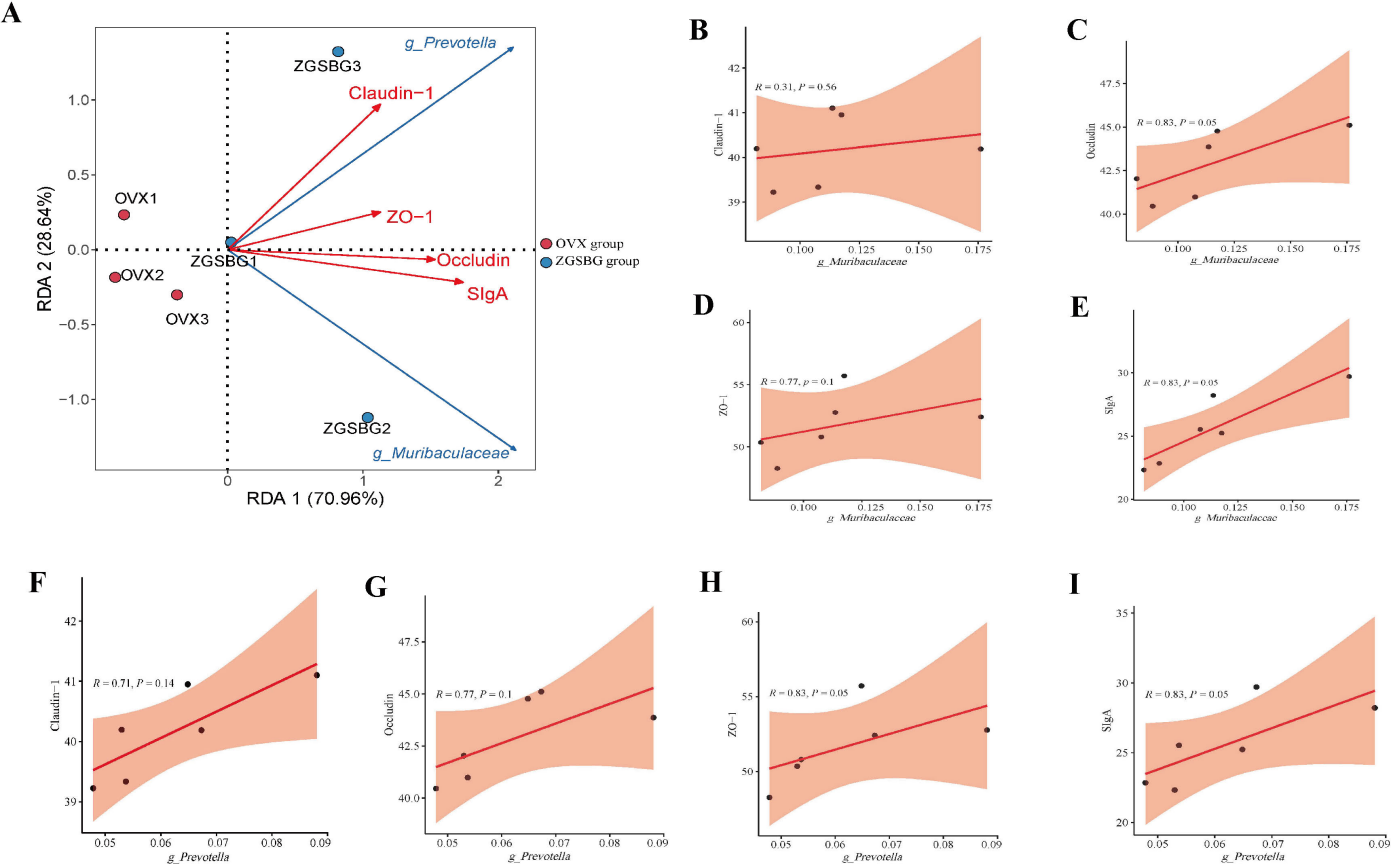
Correlation analysis between gut characteristic bacteria and indicators of intestinal mucosal barrier function. **(A)** RDA plot. **(B)** Correlation scatter plot between *Muribaculaceae* and claudin-1. **(C)** Correlation scatter plot between *Muribaculaceae* and occludin. **(D)** Scatterplot of correlation between *Muribaculaceae* and ZO-1. **(E)** Correlation scatterplot between *Muribaculaceae* and SIgA. **(F)** Correlation scatterplot between *Prevotella* and claudin-1. **(G)** Correlation scatterplot between *Prevotella* and occludin. **(H)** Correlation scatterplot between *Prevotella* and ZO-1. **(I)** Correlation scatterplot between *Prevotella* and SIgA. Sham group, sham surgery group; OVX group, ovariectomized group; ZGSBG group, Zhuanggu Shubi ointment group.

There was a positive correlation between *Muribaculaceae* and claudin-1, occludin, ZO-1, and SIgA (*P>*0.05; *P*<0.05; *P>*0.05; *P*<0.05). *Prevotella* was positively correlated with claudin-1, occludin, ZO-1, and SIgA (*P>*0.05; *P>*0.05; *P*<0.05; *P*<0.05) (**Figure 10**). Hence, there was a positive regulatory effect between the characteristic bacteria and the intestinal mucosal barrier function after the ZGSBG intervention.

**Figure 11:**
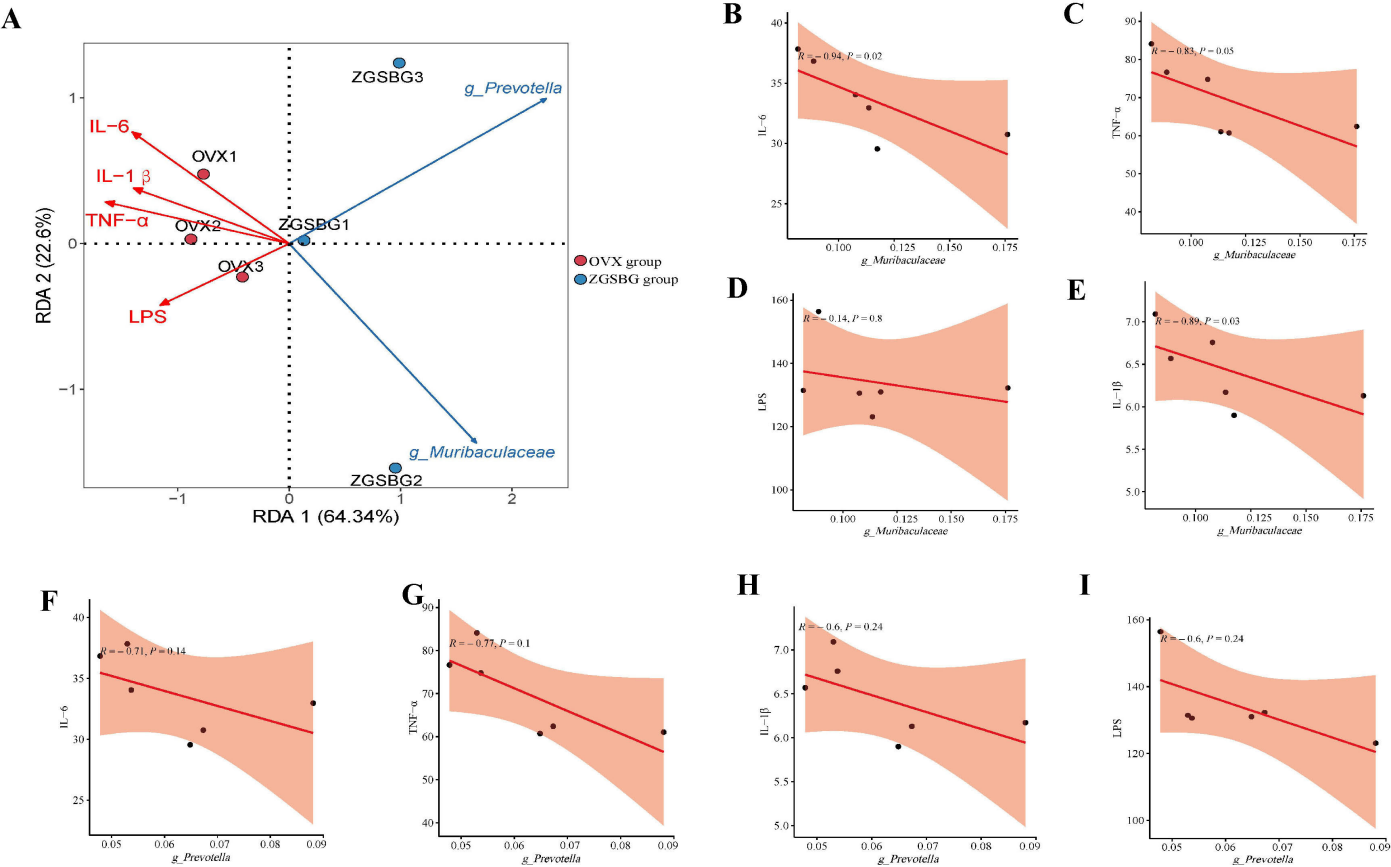
Correlation analysis between gut characteristic bacteria and indicators of inflammatory response. **(A)** RDA plot. **(B)** Correlation scatter plot between *Muribaculaceae* and IL-6. **(C)** Correlation scatter plot between *Muribaculaceae* and TNF-α. **(D)** Scatterplot of correlation between *Muribaculaceae* and IL-1β. **(E)** Scatterplot of correlation between *Muribaculaceae* and LPS. **(F)** Correlation scatterplot between *Prevotella* and IL-6. **(G)** Correlation scatterplot between *Prevotella* and TNF-α. **(H)** Correlation scatterplot between *Prevotella* and IL-1β. **(I)** Correlation scatterplot between *Prevotella* and LPS. Sham group: Sham group, sham surgery group; OVX group, ovariectomized group; ZGSBG group, Zhuanggu Shubi ointment group.

There was a significant negative correlation between *Muribaculaceae* and IL-6, TNF-α, IL-1β, and LPS (*P*<0.05; *P*<0.05; *P*<0.05; *P>*0.05). There was also a negative correlation between *Prevotella* and IL-6, TNF-α, IL-1β, and LPS (*P>*0.05; *P>*0.05; *P>*0.05; *P>*0.05) (**Figure 11**). Therefore, there was a modulating effect between the characteristic bacteria and the inflammatory response, in which *Muribaculaceae* modulated the inflammatory response more clearly.

## 4 Discussion

### 4.1 ZGSBG increased femur density, improved femur structural injury, and regulated bone metabolic balance in the PMO rats

In the experiment, we used the bilateral ovarian denervation method to replicate the PMO rat model. Combined with the changes in bone mineral density, bone histopathology, and related bone transformation indexes in serum, the ZGSBG intervention elevated the BMD, Tb.BV/TV, Tb.N, and Tb.Th levels; increased the thickness of bone trabeculae; reduced the interruption of connections between bone trabeculae; decreased the number of adipocyte cells in the bone marrow cavity; upregulated the levels of bone formation-related indicators; and downregulated the levels of bone resorption-related indicators. Thus, the efficacy of ZGSBG was obvious in increasing the bone density, improving the femur structure, and regulating the balance of bone metabolism in PMO rats.

### 4.2 ZGSBG repaired intestinal mucosal barrier damage and downregulated intestinal inflammation in the PMO rats

The intestinal mucosal barrier is the largest interface between the organism itself and the external environment and is the main line of defense of the organism against invasion by foreign antigens and pathogenic microorganisms (Li et al., 2023; Xie et al., 2023; Qiao et al., 2024). Intestinal mucosal epithelial cells are connected by tight junction proteins, which include claudin, occludin, and the ZO family (Jeong et al., 2017; Foerster et al., 2022). Among them, claudin-1 and occludin are both transmembrane backbone proteins which prevent the loss of intestinal mucosal fluid and the dispersion of macromolecules (Alizadeh et al., 2022; Dengr et al., 2018). ZO-1, as a scaffolding protein, mainly plays the role of a bridge, responsible for intercellular transport and epithelial cell polarity (Yang et al., 2020). As an effector molecule of the intestinal mucosal immune barrier, SIgA prevents pathogenic microorganisms and toxin molecules from adhering to the surface of intestinal epithelium, playing an immune role and resisting the invasion of pathogenic microorganisms on the body (Sabatino et al., 2023; Dai et al., 2022). If the intestinal mucosal barrier function is impaired, the intestinal mucosal permeability increases and intestinal bacterial translocation and pro-inflammatory factors are released in large quantities, thus aggravating the primary disease and even inducing multi-organ failure. This experiment showed that the levels of SIgA, claudin-1, occludin, and ZO-1 in the rats in the ZGSBG group were significantly higher than in the OVX group. Additionally, the levels of TNF-α, IL-1β, IL-6, and LPS were significantly lower. Thus, the ZGSBG intervention significantly repaired intestinal mucosal barrier damage and downregulated intestinal inflammation in PMO rats.

### 4.3 ZGSBG regulated the structure and function of gut microbiota in PMO rats

High-throughput sequencing has been popularized in various fields of scientific research (Liu et al., 2023; Reuter et al., 2015). Especially in the field of gut microorganisms, the rapid development of high-throughput sequencing technology has greatly facilitated and changed the knowledge of human research on the structure and function of microorganisms in the ecological environment and has led to an upsurge of gut microorganism research in the field of life sciences in recent years (Gao et al., 2021; Chen et al., 2021; Qiu et al., 2022). The intersection of multidisciplinary technologies has gradually become a major trend in scientific research (Shi and Gao, 2019; Zhou et al., 2024). In our experiments, we analyzed the gut microbiota of rats before and after modeling and drug administration with the help of bioinformatics technology. The reliability of the downstream data was analyzed before downstream analysis, which confirmed that the sample size and homogeneity of random sampling satisfied the experimental design and provided quality assurance for the subsequent analysis results. The diversity and community structure of the gut microbiota in the ZGSBG group were altered. Proteobacteria, Actinobacteriota, Campilobacterota, *Prevotella*, and *Helicobacter* might play important roles as dominant bacteria in the treatment of PMO by ZGSBG. LEfSe analysis showed that *Muribaculaceae* and *Prevotella* were enriched in the ZGSBG group, suggesting that *Muribaculaceae* and *Prevotella* might play important roles as biomarkers in the treatment of PMO with ZGSBG. Focusing on the KEGG metabolic pathways, the functional pathways of gut microbiota were enriched in PMO rats after the ZGSBG intervention, among which the GLYCOLYSIS-TCA-GLYOX-BYPASS, TCA-GLYOX-BYPASS, and PWY-7007 pathways had statistically significant differences. In summary, the intervention of ZGSBG changed the structure and function of gut microbiota in PMO rats. Characteristic bacteria *Muribaculaceae* and *Prevotella* played important roles in the treatment of PMO with ZGSBG.

### 4.4 Possible involvement of *Prevotella* and *Muribaculaceae* in intestinal mucosal barrier damage and activation of the intestinal inflammatory response during PMO

Gut microbiota are a key factor in the health of the body and the transformation of disease as they influence the physiological and pathological activities of the body by metabolizing the nutrients ingested by the body and participating in the exchange of information with the body (Parada et al., 2019; Li et al., 2014). Inflammation is a double-edged sword for the health of the body. Inflammation is important for the body’s own defense, but excessive or persistent systemic inflammation can have adverse effects (Casanova and Abel, 2021). Research has proved that there is a tight and complex regulatory network between gut microbiota and the inflammatory response (Zhang et al., 2022; Zhu et al., 2022). Gut microbiota and the bioactive molecules they produce interact with immune cells and participate in the body’s inflammatory process. The disturbance of gut microbiota damages the intestinal mucosal barrier, leading to the release of LPS, activating local and systemic immune responses, and releasing excessive inflammatory factors (Guan et al., 2021). Inflammatory factors are pathophysiological mediators in the inflammatory process. Inflammatory factors including IL-6, TNF-α, and IL-1β are involved in the growth, differentiation, and functional regulation of a variety of tissue cells in the organism, and they are the main pathogenic inflammatory factors in the inflammatory response of the organism (Zhu and Ma, 2021). Combined with the results of this experiment, *Prevotella* and *Muribaculaceae* might act as biomarkers to influence the ZGSBG treatment in PMO. Among them, *Prevotella* is a gram-negative bacterium that belongs to the Mycobacterium phylum and improves the absorption and utilization of nutrients, which are closely related to the metabolism of the host organism (Zhao et al., 2023). Zhang et al. revealed that *Prevotella* was closely related to intestinal mucin synthesis, and its reduced abundance impaired intestinal mucosal barrier function, increased intestinal permeability, promoted the entry of toxic substances into the bloodstream, and then induced systemic inflammation. The current reports on *Muribaculaceae* mainly focus on its positive role as a beneficial bacteria in disease treatment. The enrichment of *Muribaculaceae* in the intestine inhibited the release of LPS into the blood and alleviated intestinal mucosal barrier dysfunction, inflammatory response, and lipid metabolism disorders caused by acute myocardial infarction (Zhong et al., 2023). The positive regulatory effects of *Prevotella* and *Muribaculaceae* on intestinal mucosal barrier function and inflammatory response have been demonstrated. In our experiment, this was also confirmed by correlation analysis, i.e., *Muribaculaceae* and *Prevotella* were negatively correlated with IL-6, TNF-α, IL-1β, and LPS, and positively correlated with SIgA, claudin-1, occludin, and ZO-1. In summary, we hypothesize that ZGSBG might repair intestinal mucosal barrier damage and downregulate intestinal inflammation during PMO by enriching the characteristic bacteria *Muribaculaceae* and *Prevotella*.

## 5 Conclusion

**Figure 12:**
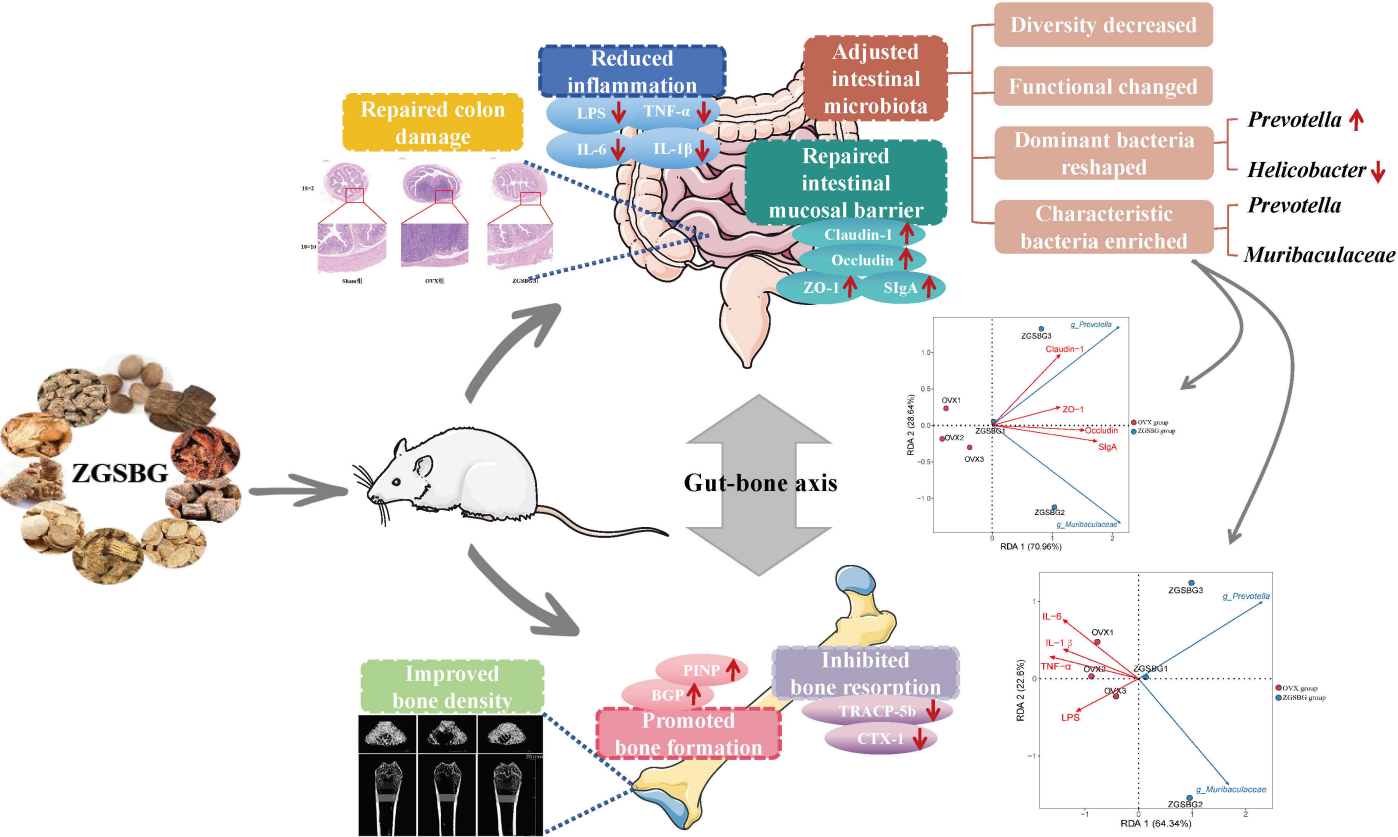
Gut microecological mechanism of ZGSBG in the treatment of postmenopausal osteoporosis.

In this study, ZGSBG regulated bone density, promoted bone formation, inhibited bone resorption-related indexes, and restored the structural damage of femurs, thus achieving the effect of treating PMO. The mechanisms of action of ZGSBG in treating PMO were found to be by regulating gut microbiota, repairing intestinal mucosal barrier damage, and downregulating the level of intestinal inflammation. The characteristic bacteria *Muribaculaceae* and *Prevotella* might play active roles in the process of intestinal mucosal barrier damage and inflammatory response occurrence in PMO treated with ZGSBG (**Figure 12**).

## Data Availability

The datasets presented in this study can be found in online repositories. The names of the repository/repositories and accession number(s) can be found below: https://www.ncbi.nlm.nih.gov/, PRJNA1031800.
